# Evidence for microRNA-31 dependent Bim-Bax interaction preceding mitochondrial Bax translocation during radiation-induced apoptosis

**DOI:** 10.1038/srep15923

**Published:** 2015-10-30

**Authors:** Ashish Kumar, Soma Ghosh, Sudhir Chandna

**Affiliations:** 1 Natural Radiation Response Mechanisms Group, Division of Radiation Biosciences, Institute of Nuclear Medicine & Allied Sciences, Brig. S.K. Mazumdar Road, Timarpur, Delhi-110054, India

## Abstract

Downregulation of microRNA-31 has been linked with enhanced stress resistance, while its overexpression leads to cell death. In this study, we found mediatory role of microRNA-31 in γ-radiation-induced apoptosis in a model insect cell line Sf9 carrying well-conserved apoptotic machinery. Mature microRNA-31 is perfectly conserved amongst insects; hence we used biotinylated probes designed from *Bombyx mori* sequence for its successful detection in Sf9 cells. Target identification using *Bombyx mori* 3′UTRs predicted miR-31′s potential role in Lepidopteran apoptosis, which prompted us to investigate alterations in its expression during radiation-induced cell death. We found significant overexpression of Sf-miR-31 following lethal dose (1,000Gy–3,000Gy) irradiation. Its mediatory role was finally confirmed as antisense-microRNA-31 could successfully inhibit radiation-induced cytochrome-c release, caspase-3 activation and apoptosis. While Bax/Bcl-2 expression remained unchanged, lethal radiation doses induced Bim overexpression and direct Bim-Bax interaction (co-immunoprecipitation) which is not yet unequivocally demonstrated during apoptosis. Quite important, these events were found to be dependent on radiation-induced miR-31 overexpression, as antisense-miR-31 inhibited both the responses and resulted in significant inhibition of cell death. Pro-apoptotic role of miR-31 was further confirmed when miR-31 mimic induced apoptosis involving similar Bim/Bax alterations. Therefore, our study reveals an important mediatory role of miR-31 in radiation-induced cell death.

MicroRNAs (miRNAs) are evolutionarily conserved 20–22 nucleotide long non-translating RNAs that are transcribed by RNA polymerase-II and comprise about 1–3% of the genome^1^,^2^,^3^. Biogenesis of miRNAs involves multistage processing spread over nuclear as well as cytoplasmic compartments, and culminates in inclusion of 20–22 nucleotide long single strands in a protein complex known as RNA-induced silencing complex (RISC) mainly comprising of Argonaute-2 (AGO2). This miRNA-induced silencing complex (miRISC) finally mediates translational repression and/or increased degradation of its mRNA targets^4^ by binding to 3′ UTR’s. A single miRNA may be able to repress multiple different transcripts and hence regulate multiple pathways and responses by altering protein expression. Therefore, miRNA’s are now recognized as important cell fate determinants in response to cellular environment as well as exposure to chemical agents or radiation.

Radiation-induced alterations in the expression level of certain miRNAs have been demonstrated in various cell types^5^,^6^,^7^. Increased expression of some miRNAs like miR-1285, miR-151-5p, Let-7i may enhance radioresistance while Let-7 upregulation has been shown to increase radiosensitivity in various cancer cell lines following clinical dose (~2Gy X-Rays) irradiation^8^. However, limited studies have so far been conducted on the role of miRNAs in cellular radiosensitivity, and many miRNAs remain to be investigated. Out of various miRNAs now known for regulating cellular functions, we aimed to study miRNAs that carry substantially high specificity while having good pleiotropic activity. One such miRNA is miR-31, which is known to regulate a variety of cellular functions. Interestingly, miR-31 is the only known miRNA that is present in vertebrates as well as in *Drosophila* and carries an eight nucleotide motif (seed region) that squarely enhances its targeting specificity to a level unmatched by most other miRNA’s^9^. It has been found significantly downregulated in many cancerous cells^10^,^11^,^12^ while its upregulation is linked with metastatic regression and increased cell death^13^,^14^. It has also been shown to target genes involved in apoptosis regulation including E2F6^15^, Receptor tyrosine kinase MET^16^, Protein kinase-C epsilon^14^, NF-κB inducing kinase (NIK)^17^, fibroblast growth factor 3 (FGF3)^18^, and pathways like Akt-dependent signaling as well as Bim induction^13^. In an isolated study, down-regulation of miR-31 was shown to be associated with increased radioresistance^19^, although underlying mechanism is not yet known. Therefore, miR-31 is an important candidate for studying its role in cellular radioresistance/radiosensitivity.

In the present study, we investigated whether altering the miR-31 level can modify cellular radioresistance, using a cell system that efficiently expresses this specific miRNA. We chose a model cell system (Sf9 ovarian cell line derived from *Spodoptera frugiperda*; class *Insecta*, order Lepidoptera) that shows unusual radioresistance (~100–200 times higher than human/mammalian cells^20^) marked by excessive resistance against radiation-induced apoptosis^20^,^21^. Due to such level of resistance achieved despite extensive structural/functional homologies with mammalian cells, it is considered an important higher eukaryotic model system to study molecular radiation responses. High radioresistance of Sf9 cells allows choice of fairly distant sub-lethal versus lethal doses for delineating the molecular pathways elicited during cell death. Factors involved in radioresistance of these cells include unusually low induction of DNA damage and oxidative stress^22^, efficient DNA repair^23^,^24^ absence of nitrosative stress^25^, and higher resilience to death-inducing signals^21^,^26^. Very little is however known about the miRNA-mediated regulation of apoptosis in these cells^27^. In the present study, we not only detected the existence of miR-31 in Sf9 cells, but also found its significant contribution in mediating radiation-induced cell death through a novel molecular pathway. Our study also suggests that downregulation of miR-31 could contribute to cellular radioresistance, and hence adds significantly to the understanding of cellular radiation responses.

## Results

### Identification of Sf-miR-31 and predicting its putative physiological role

**Figure 1:**
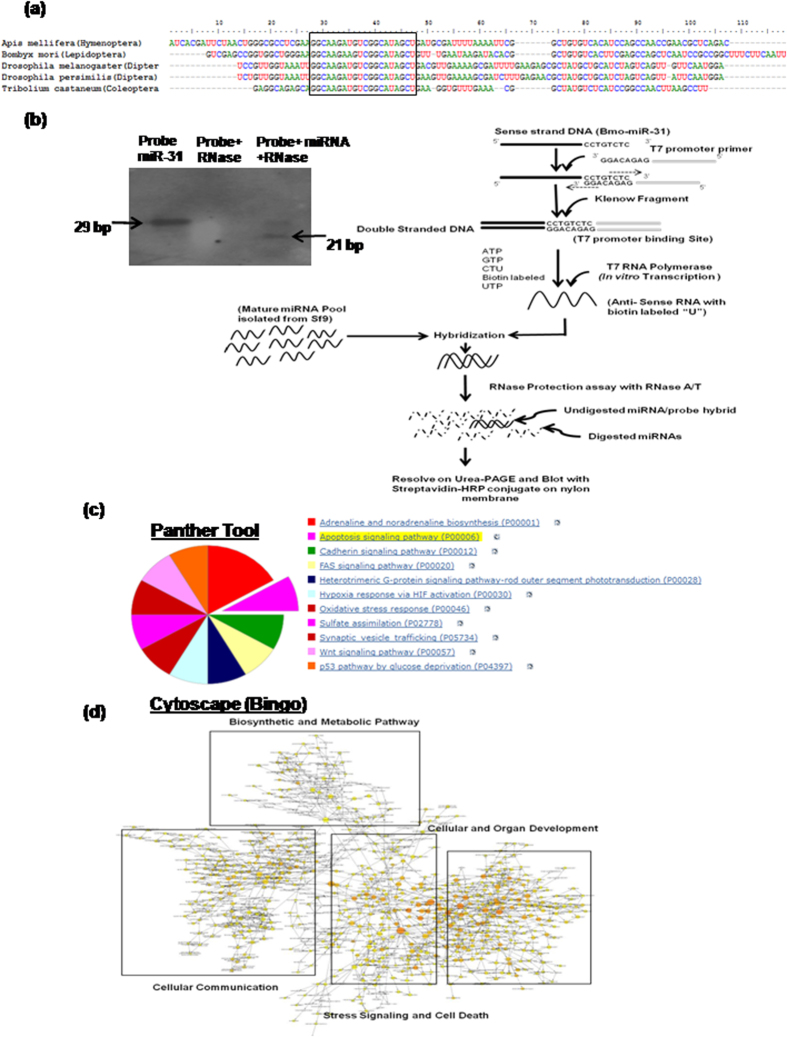
Identification and functional annotation of miRNA-31 in Sf9 cells. (**a**) Clustal W analysis of miR-31 sequences from five different species of the same order shows miR-31 conservation at mature miRNA level; (**b**) As shown in the schematic, *in vitro* transcribed biotinylated probes were used with miRNAs isolated from Sf9 cells for RNase protection assay followed by chemiluminescent blotting (with streptavidin- HRP conjugate) for the identification of miR-31. First lane on the left shows the *in vitro* transcribed probe. Middle lane shows the probe digested with RNase. In the right lane, upper band shows undigested probe and lower band depicts 21 bp mature miR-31; (**c**) Sf-miR-31 sequence was queried against all the predicted UTRs of Bombyx mori and the predicted potential targets were mapped using panther tool for analysis of molecular functions and biological processes. Predicted targets of Sf-miR-31 majorly include regulatory pathways of apoptotic signaling, among few other important cellular functions. (**d**) Cytoscape (Bingo) tool was used for Gene Ontology term clustering of all the predicted targets. The four major biological pathways predicted as regulated by Sf-miR-31 are shown in different groups within the hollow boxes.

Fully annotated nucleic acid sequences or microRNA database for *Spodoptera* are not available. Therefore, we extracted pre-miRNA-31 sequences from different orders of class *Insecta* using the microRNAs database (miRbase.org). ClustalW analysis of these pre-microRNA-31 sequences showed near–complete conservation within the mature microRNA-31 (miR-31) region across various orders including Lepidoptera (Fig. 1a). It was thus apparent that this conservation in the mature microRNA region should be definite within the same order. To test this, we used *Bombyx mori* miR-31 (order Lepidoptera) as a reference sequence for generating biotinylated probes for Sf9 (Fig. 1b). Hybridization of biotinylated probes with the miRNA pool isolated from Sf9 cells followed by RNase protection assay showed an integral single band (Fig. 1b), confirming complete homology of mature miRNA-31 between the two Lepidopteran species, viz., *Bombyx mori* and *Spodoptera frugiperda* as suggested previously by Mehrabadi, M. *et al*.^28^.

Target prediction for miR-31 using PITA executable software showed many important genes that are important for stress signaling, cell death, cellular and organelle development as well as cellular communication (Supplementary Table 1). Gene Ontology (GO) term clustering of Bmo-/Sf-miR-31 using *Panther* and *Cytoscape (Bingo)* tools suggested involvement of Sf-miR-31 in diverse cellular processes (Fig. 1c,d). Cell death/apoptosis regulation and stress signaling are among the important predicted target pathways of Sf-miR-31. These *in-silico* predictions hence strongly suggest Sf-miR-31 as a regulator of cell death/apoptosis.

### Radiation-induced caspase-3 dependent cell death coincides with Sf-miR-31

**Figure 2:**
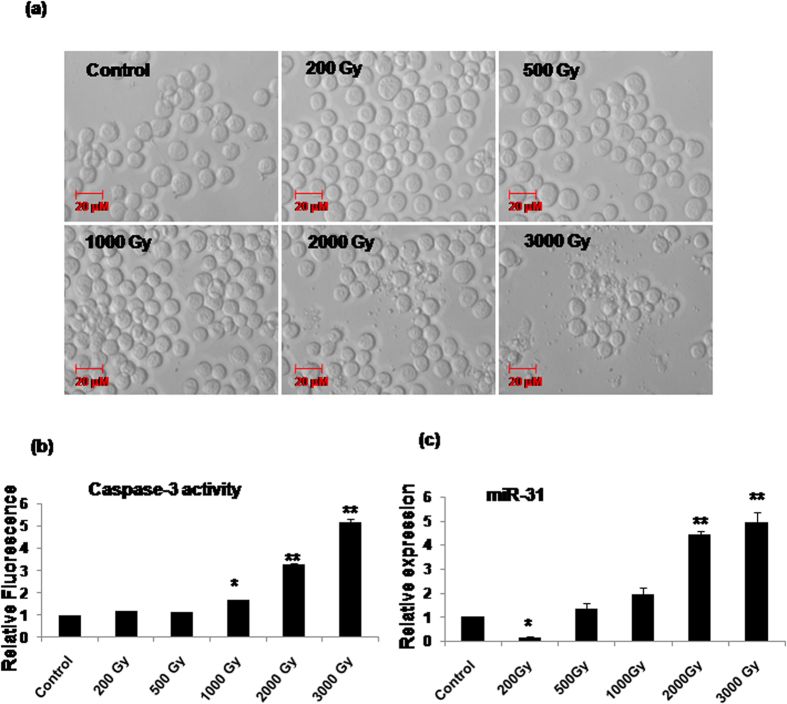
Alteration in Sf-miR-31 expression corresponds with radiation-induced caspase activation and apoptosis. (**a**) DIC microscopy images of Sf9 cells 24h following irradiation with different doses show presence of apoptotic bodies in cultures irradiated at 2000Gy–3000Gy. Images are representative of three independent experiments. (**b**) Sf9 cells were irradiated at different doses and lysed for caspase-3 activation assay. Doses ≥1000Gy induce significant caspase-3 activation in a dose-dependent manner. Data are mean ± SD of three independent experiments. (*P < 0.001, **P < 0.02); (**c**) Real Time quantitative PCR analysis shows alterations in Sf-miR-31 expression 24 h after irradiation at different doses. Expression reduced significantly at 200Gy whereas it increased in a dose-dependent manner at 1000Gy-3000Gy. Data are mean ± SD of three independent experiments (*P < 0.005, **P < 0.002).

We have earlier shown that doses up to 200Gy fail to induce significant apoptosis in Sf9 cells^20^. In this study as well, Sf9 cells irradiated with 200Gy and 500Gy of γ-radiation did not undergo caspase-3 activation or apoptosis (Fig. 2). Interestingly, the Sf-miR-31 expression was significantly downregulated at these sub-lethal doses (p < 0.05). On the other hand, a dose-dependent increase in caspase-3 activation and cell death marked by typical apoptotic bodies (Fig. 2a,b) was evident by 24 h following irradiation at higher doses (1,000Gy–3,000 Gy), which was associated with a concomitant increase in the expression of Sf-miR-31 (p < 0.002; Fig. 2c).

### Suppression of Sf-miR-31 inhibits radiation-induced cell death

**Figure 3:**
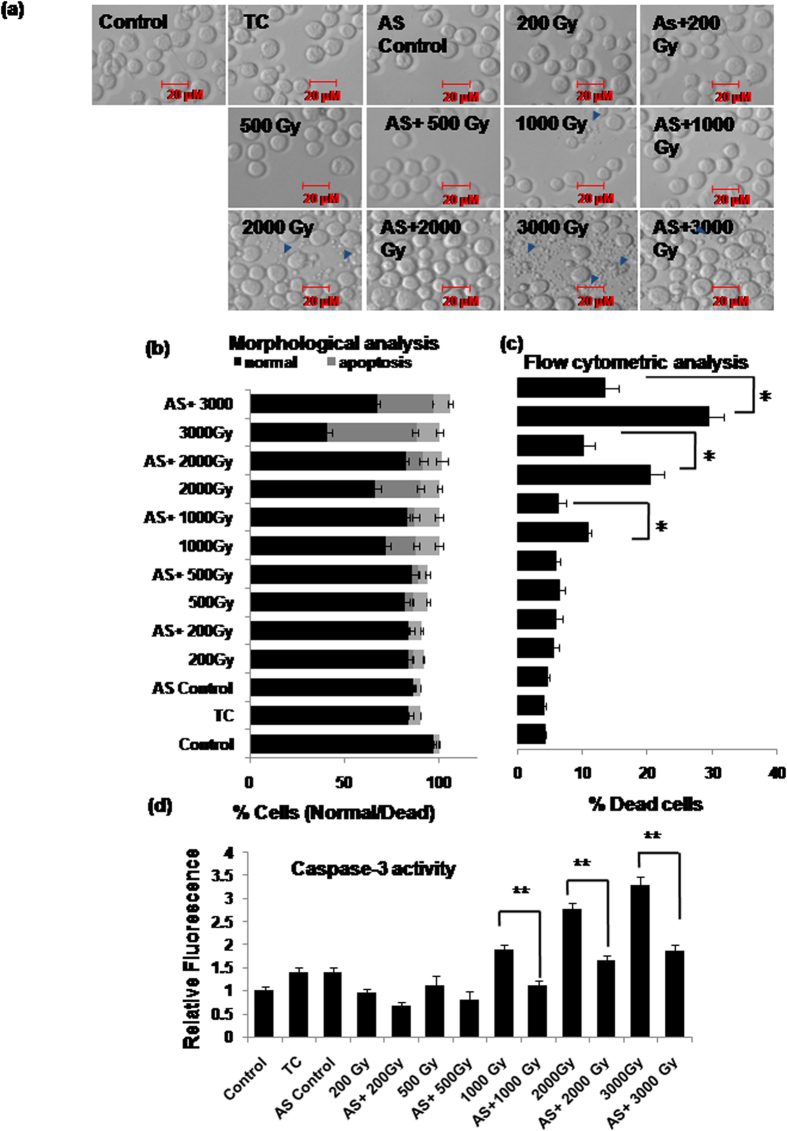
Inhibition of miR-31 decreases caspase-dependent Sf9 apoptosis. (**a**) Sf9 cells were transfected with AS-miR-31 and irradiated at different doses 4–6 h later. Significant reduction in apoptosis is evident after Sf-miR-31 inhibition using antisense RNA at doses 2000Gy-3000Gy (16h–20 h post irradiation). Transfection control (TC) and scrambled antisense control (ASC) were used to confirm the specific effects of antisense-miR-31. Arrows depict apoptotic bodies. Images are representative of three independent experiments. (**b**) Fluorescence-based morphological analysis of harvested cells shows inhibition of radiation-induced cell death by AS-miR-31. Data are mean ± SD of three independent experiments. (**c**) Flow cytometric analysis of harvested cells shows inhibition of radiation-induced cell death by AS-miR-31. Data are mean ± SD of three independent experiments. (*P < 0.05) (**d**) Caspase-3 activity was also reduced in lethally irradiated cells by pre-transfection with AS-miR-31. Data are mean ± SD of three independent experiments. (**P < 0.02).

In order to confirm whether overexpression of Sf-miR-31 has a functional role in the induction of radiation-induced Sf9 cell death, its expression was suppressed using antisense RNA prior to irradiation, and alteration in cell death induction was studied using morphological scoring (necrotic/apoptotic cells), flow cytometry (sub-G1 population) as well as by caspase-3 activity. Quite important, radiation-induced overexpression of Sf-miR-31 at doses >1,000Gy resulted in significant inhibition (p < 0.05) of cell death when it was inhibited/compromised using antisense miR-31 (AS-miR-31) (Fig. 3).

### Radiation induced Bax translocation in the absence of Bax/Bcl-2 alterations is mediated by miR-31 overexpression

**Figure 4:**
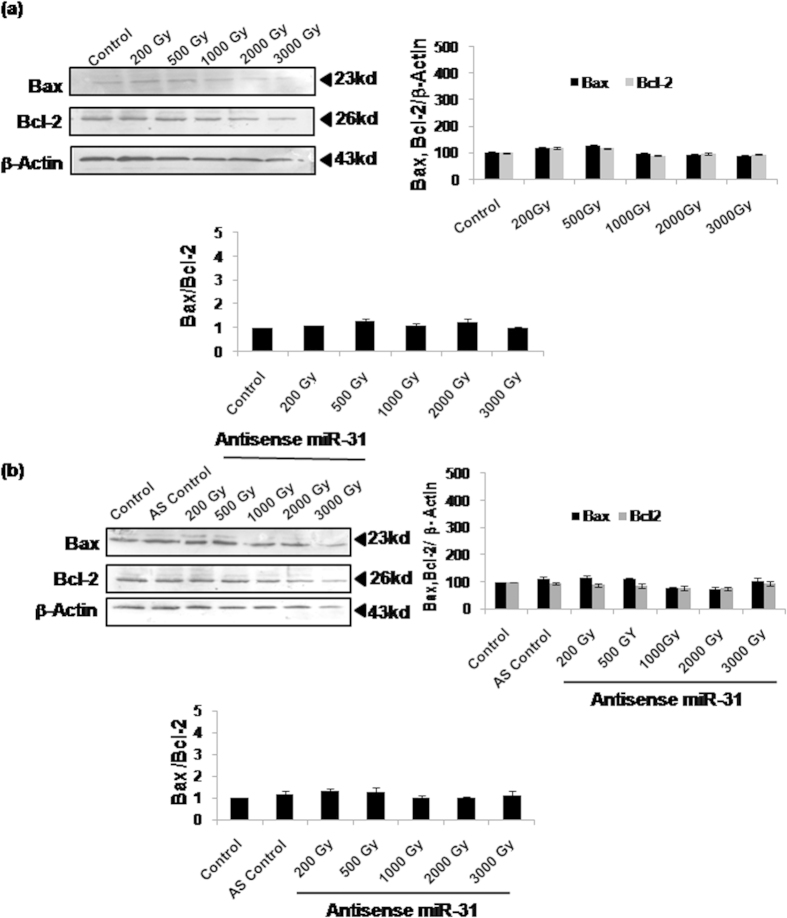
Bax and Bcl-2 expression/ratio remains unaltered following irradiation or AS-miR-31 transfection. (**a**) Densitometry of western immunoblots of Bax and Bcl-2 16 h post-irradiation failed to show any change in the Bax or Bcl-2 expression levels or in Bax/Bcl-2 ratio. (**b**) Similarly, the expression or ratio of Bax/Bcl-2 remained unchanged following pre-treatment with AS-miR-31 prior to irradiation. Immunoblot images are representative of three independent experiments yielding similar results.

**Figure 5:**
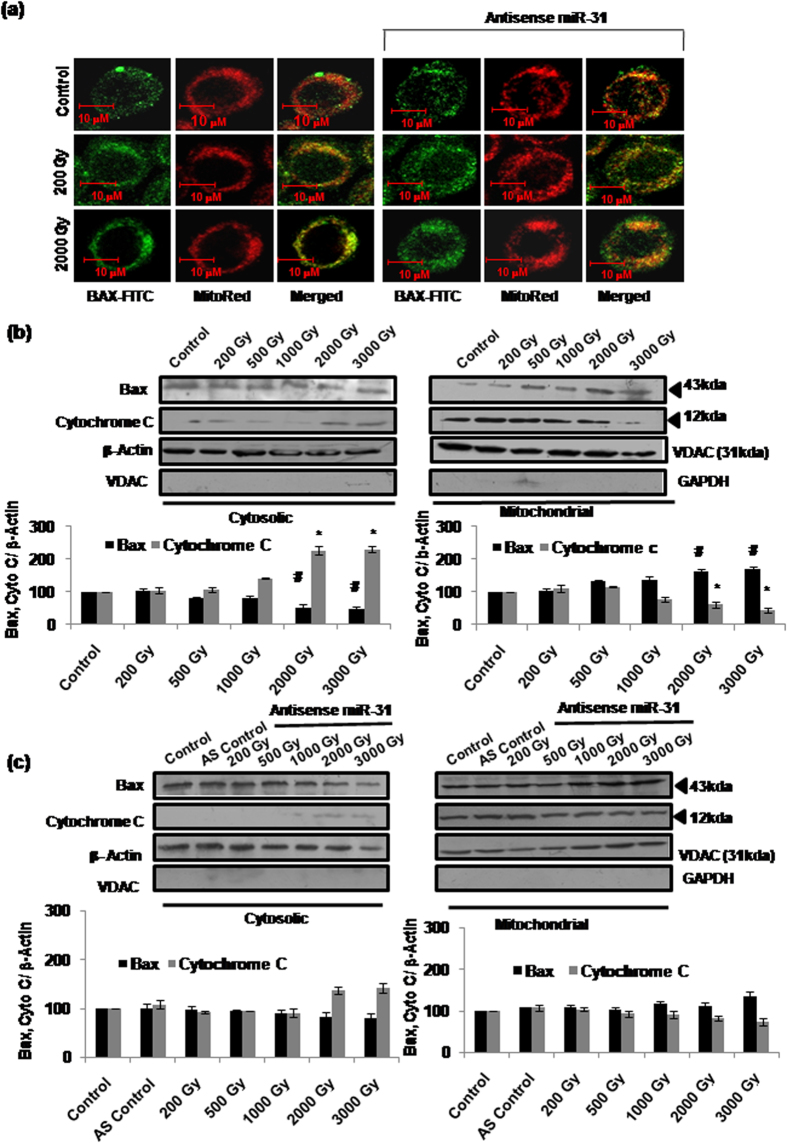
miR-31 dependent translocation of Bax from cytosol to mitochondria corresponds with cytochrome-c release. (**a**) Sf9 cells were grown on cover slips and irradiated with or without prior transfection with As-miR-31. Bax protein was tagged using Anti-Bax-FITC antibody conjugate and mitochondria were counterstained with MitoRed. Merged signals confirm the translocation of Bax to mitochondria. Sub-lethal (200Gy) and lethal (2000Gy) radiation doses were used with and without prior treatment with AS-miR-31. Images were captured using 63 × 1.4 NA objective (Leica Sp8 spectral confocal imaging system). Images are representative of four independent experiments. (**b**) Translocation of Bax to mitochondria (and of cytochrome-c into cytosol) was further assessed by subcellular (cytoplasmic and mitochondrial) fractionation and western immunoblotting at 16 h post-irradiation (^#^,*P < 0.05). Purity of cytosolic and mitochondrial fractions was confirmed using anti-VDAC and anti- GAPDH antibodies, respectively. (**c**) Cytosolic and mitochondrial fractions transfected with AS-miR-31 were also used for the western blot analysis for Bax translocation and cytochrome-c release. While β-actin was used as standard loading control for cytosolic fraction, VDAC was taken as loading control for the mitochondrial fraction.

Recent study from our laboratory has established that radiation induces cell death in Sf9 cells in a primarily Bax-dependent manner^21^. In the present study, we further observed that expression of pro-apoptotic protein Bax or anti-apoptotic protein Bcl-2 is not altered by radiation doses (1,000Gy–3,000Gy) inducing apoptosis (Fig. 4a). Pre-treatment with AS-miR-31 also failed to alter Bax and Bcl-2 expression/ratio (Fig. 4b). Despite unchanged protein expression, we found clear evidence of translocation of Bax to mitochondria following irradiation at 1,000Gy–3,000Gy, using immunofluorescence microscopy (Fig. 5a) as well as by immunoblotting of mitochondrial and cytosolic fractions (Fig. 5b). Inhibition of Sf-miR-31 using antisense significantly inhibited Bax translocation induced by high doses, which implied an apparently indirect regulation of Bax-mediated processes by miR-31 (Fig. 5c,a). Lethal doses also caused release of cytochrome-c from mitochondria into cytosol following irradiation at 1,000Gy–3,000Gy and inhibiting Sf-miR-31 resulted in its partial but significant reduction (Fig. 5c). These results thus show that Bax translocation and consequent cytochrome-c release/cell death are partly regulated by Sf-miR-31.

### Radiation-induced miR-31 overexpression enhances Bim expression as well as its interaction with Bax

**Figure 6:**
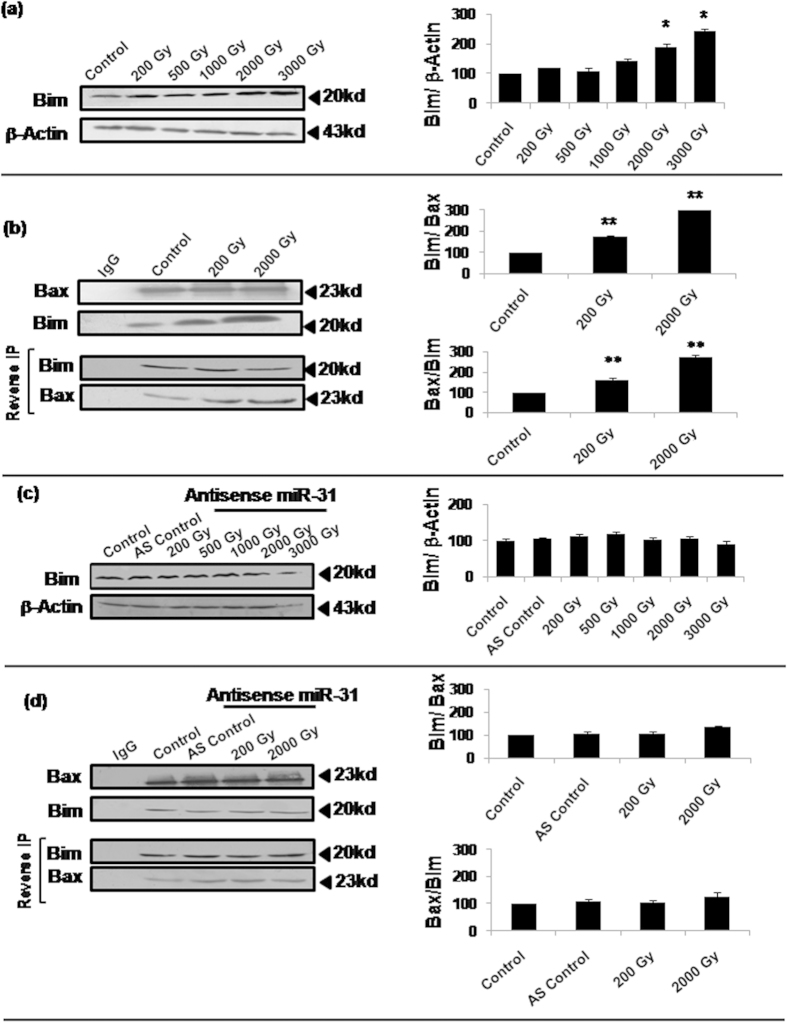
Bim overexpression and its increased interaction with Bax was associated with Bax translocation to mitochondria. (**a**) Western blot analysis of Bim was performed 16h post-irradiation for all the selected doses. Densitometric analysis showed a dose-dependent increase in Bim expression (*P < 0.002). (**b**) Co-immunoprecipitation western immunoblots show highly significant increase in the interaction of Bim with Bax (**P < 0.02). Sf9 cells were irradiated, harvested 16h later, lysed in native condition, followed by immunoprecipitation by anti-Bax antibody. The immunoprecipitated samples were then used for immunodetection of Bim interacting directly with Bax. For further confirmation of Bim –Bax interaction, reverse immunoprecipitation was carried out using anti-Bim antibody and detection by anti-Bax antibody (labeled as ‘Reverse IP’). We chose sub-lethal (200Gy) and lethal (2000Gy) radiation doses for assessing differences in Bim-Bax interaction, and IgG alone was used as antibody control (**P < 0.02). Densitometric analysis was done for the quantitation of all immunoblots. (**c**,**d**) Radiation-induced alterations in the expression of Bim as well as its interaction with Bax were also observed after inhibition of Sf-miR-31 by AS-miR-31. Using same radiation doses as above samples were processed for co-immunoprecipitation as well as reverse immunoprecipitation.

We further explored potential regulators of radiation-induced Bax translocation to mitochondria. Bim (and its BH3-only peptide) has been earlier shown to interact with Bax^29^,^30^, although consequent effect on Bax functioning was not clearly shown. We investigated whether Bax translocation to mitochondria involves Bim-Bax interaction following lethal dose irradiation. Western immunoblotting confirmed a dose-dependent increase in Bim expression especially at doses >1,000Gy (Fig. 6a). Co-immunoprecipitation experiments further revealed direct interaction of Bim and Bax that was considerably increased at lethal radiation doses inducing mitochondrial translocation of Bax (Fig. 6b). In these experiments, we performed immunoprecipitation (IP) of either proteins (IP and reverse IP) followed by detection of the interacting protein.

Since AS-miR-31 was able to significantly inhibit the induction of Bax translocation as well as apoptosis, we tested if radiation-induced Bim overexpression is also altered in this condition. As shown in Fig. 6c,d, treatment with AS-miR-31 indeed caused inhibition of radiation-induced Bim overexpression and Bim/Bax interaction, confirming miR-31’s role in regulating Bim-Bax pathway.

### Ectopic overexpression of miR-31 induces apoptosis in Sf9 cells and confirms its mediatory role in cell death

**Figure 7:**
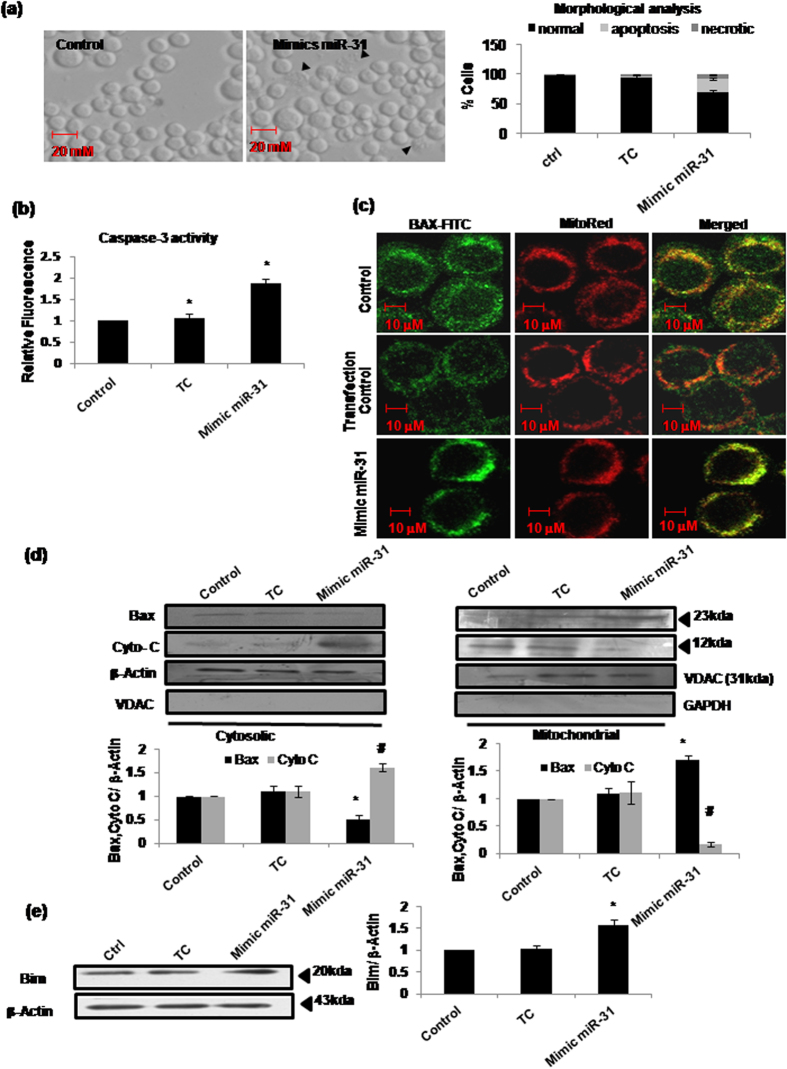
Ectopic overexpression of Sf-miR-31 using miR-31 mimic sequence induced significant cell death in a Bim-Bax dependent manner. (**a**) Sf-miR-31 mimic oligos used for overexpression of miR-31 induced apoptosis in Sf9 cells as evident in the morphological analysis 24h after transfection. Arrow heads in the DIC images mark apoptotic bodies. (**b**) Caspase-3 activation assay also suggests significant induction of cell death (*P < 0.05). Transfection control (TC) was also used to test the sensitivity of Sf9 cells to the transfection reagent. (**c**) Significant translocation of Bax to mitochondria was evident using immunofluorescence microscopy and (**d**) western immunoblotting, 18h after transfection with miR-31 mimics (^#,*^P < 0.02). Purity of cytosolic and mitochondrial fractions was confirmed using anti-VDAC and anti- GAPDH antibodies, respectively. (**e**) Significant increase was observed in Bim expression 18h after transfection with miR-31 mimics (* P < 0.02).

Since radiation induced significant overexpression of miR-31 which in turn mediated Bim-Bax pathway, we further confirmed the pro-apoptotic role of miR-31 by ectopically enhancing its expression in the unirradiated cells. Mere transfection of Sf9 cells with the miR-31 mimic sequence oligos could induced apoptotic cell death. Approximately four-fold increase in miR-31 level (using real-time PCR) following transfection with miR-31 mimics resulted in ~20% increase in cell death (Fig. 7a), which was associated with increase in caspase-3 activity (Fig. 7b) as well as significant mitochondrial Bax translocation and cytochrome-c release(^*,#^P < 0.02) (Fig. 7c,d). Importantly, miR-31 mimic-induced apoptosis also involved the upregulation of Bim expression (Fig. 7e).

## Discussion

This study presents significant evidence to demonstrate that microRNA-31, which has been recently reported to regulate tumour radiosensitivity^19^, is a prominent mediator of radiation-induced cell death in higher eukaryotes. Even more important, the study demonstrates that radiation-induced miR-31 alters Bim-mediated regulation of Bax translocation that in turn results in the post-irradiation mitochondrial cytochrome-c release and apoptosis. We used a model cell system that offers very high radioresistance and thereby allows choice of fairly distant sub-lethal versus lethal doses. These cells also carry a well conserved apoptotic mechanism that is highly homologous to the human system. In fact, we had recently reported that Sf9 cells undergo Bax-dependent apoptosis that is similar to the mitochondria-mediated intrinsic pathway known in human/mammalian cells^21^. Since miR-31 is also known to be highly conserved from insects to mammals and regulates many cellular responses/functions, the present study provides a valuable insight into the potential role of this microRNA in the cellular radiosensitivity of higher eukaryotes.

We detected miR-31 in Sf9 cells using *in silico* and experimental steps that involved *in vitro* transcription and generation of biotinylated probe, despite the fact that complete transcriptome database was not available for the organism *Spodoptera* including the miRNA sequences. Since our *in silico* analysis showed that mature miR-31 sequence is perfectly conserved between as well as within different insect orders (Fig. 1a), we used the *Bombyx mori* sequence for designing these biotinylated probes (as detailed in the methods section) for detecting miR-31 in the RNA pool of Sf9 cells. Functional annotation analysis further predicted participation of Sf-miR-31 in various cellular functions including the regulation of apoptosis (Fig. 1c,d; Supplementary Fig. 3 and Supplementary table 1). Following this, our results showing upregulation of miR-31 in cells irradiated at lethal doses indeed indicated its mediatory role in apoptosis, which was consequently confirmed by significant alteration (inhibition) of cell death by the ectopically administered AS-miR-31(*P < 0.05, **P < 0.02) (Fig. 3). Pro-apoptotic role of miR-31 is further clear from the fact that at sub-lethal doses (<500Gy), its expression was significantly downregulated (Fig. 2c). Together, our results confirm the existence of miR-31 as well as its mediatory role in radiation-induced cell death in the radioresistant Sf9 cells.

While our recent study indicated obligatory involvement of Bax in the radiation-induced apoptosis in Sf9 cells^21^, the present findings further show translocation of cytosolic Bax to mitochondria as a critical step. Studies in mammalian/human cells have generally shown that Bax/Bcl-2 ratio is increased significantly during mitochondria-mediated apoptosis^31^,^32^,^33^ largely as a result of induced Bax overexpression^32^,^34^,^35^,^36^, even as some contradictory reports suggest lack of Bax overexpression during apoptosis involving its translocation^37^,^38^,^39^. In our study, Bcl-2 or Bax expression (thereby Bcl-2/Bax ratio) failed to show any alteration at any of the lethal radiation doses used (Fig. 4a), even as Bax translocation was quite evident (Fig. 5a,b). This prompted us to further investigate the possible mechanism underlying Bax translocation at these high radiation doses. In a recent study^29^, binding of Bax with the BH3-only peptide of Bim was shown. Although Bim has not been directly implicated in the mitochondrial translocation of Bax, this remote observation encouraged us to investigate whether Bim-Bax interaction occurs constitutively or following irradiation. Our co-immunoprecipitation results (Fig. 6b) indeed show direct Bim-Bax interaction that is enhanced by lethal radiation doses involving Bax translocation. We further found that this radiation-induced Bim-Bax interaction is effectively inhibited by AS-miR-31, and so is the radiation-induced Bim overexpression (Fig. 6c,d). An isolated report has earlier shown upregulation of Bim by miR-31 albeit in unirradiated cells^13^. Therefore, Bim upregulation may be consequently induced through miR-31 overexpression in different stress responses.

Since microRNAs invariably seem to suppress the expression of their putative targets, miR-31 may target an unknown transcript which in turn negatively regulates the expression of Bim under constitutive condition. This indirect relationship between miR-31 and Bim was further corroborated when we treated Sf9 cells with the mimic sequences of miR-31. A nearly four-fold increase in miR-31 expression was sufficient to cause ~20% increase in apoptotic cell death, which was essentially mediated through Bim overexpression, Bax translocation and cytochrome-c release into cytosol (Fig. 7). Therefore, our study confirms beyond doubt that miR-31 overexpression mediates Bax-dependent cell death through indirect induction of Bim overexpression by suppressing an intermediate gene/protein. We are presently looking for these putative targets of miR-31 that could be regulating Bim expression. *In silico* predictions indicate few proteins (e.g., BG4, LKb1, MKK4, EGFR and Jafrac1) that seem important in the regulation of apoptosis (Supplementary Fig. 3 and table 1). However, the possibility of missing some additional proteins cannot be ruled out as all the predicted 3′UTR targets cannot be traced to specific genes due to lack of completely annotated *B. mori* genome sequence. Further, administering AS-miR-31 could inhibit radiation-induced cell death significantly but partially (Fig. 3). Therefore, it cannot be an exclusive cell death pathway and additional mechanisms also seem to contribute in the radiation-induced cell death.

To summarize, our study reveals a hitherto unknown cell death regulation mechanism involving two significant features. Firstly, the well-known Bax translocation to mitochondria induced by lethal radiation doses was regulated by Bim overexpression as well as direct Bim-Bax interaction in the absence of alterations in Bax or Bcl-2 expression. Secondly, these radiation-induced alterations in Bim-Bax ultimately leading to Bax translocation were dependent on miR-31 upregulation through yet unknown mediators. Interestingly, this study also implies that miR-31 downregulation could result in enhanced cellular radioresistance, since sub-lethal radiation doses were associated with considerable reduction in its expression level. Even as further investigations may be needed to thoroughly elucidate the mechanisms, our study demonstrates definite role of miR-31 in radiation-induced cell death.

## Materials and Methods

### Cell line and irradiation

Sf9 cell line, originally established from the ovaries of *Spodoptera frugiperda* (obtained from National Institute of Immunology, New Delhi, India), was maintained as semi-adherent monolayer in the 25 cm^2^ culture flasks (Falcon, BD Biosciences, Cat. No. 353108) at 28 °C in Grace’s insect cell medium (Sigma, Cat. No. G9771) supplemented with 3.33 g/l lactalbumin hydrolysate (Sigma, Cat. No. V196623), 3.33 g/l yeastolate (BD Biosciences, Cat. No. 255772), 0.35 g/l NaHCO_3_ and antibiotics (Penicillin sodium salt 50,000 units/l, streptomycin sulfate 50,000 μ g/l, Nystatin 2000 μ g/l from 500,000 USP Units/mg; Sigma). Growth medium (pH 6.2) was prepared by adding 10% heat inactivated fetal bovine serum (FBS) (Sigma, Cat. No.10082-147). Cells were subcultured twice a week in the exponential phase by seeding at a density of 35,000–40,000 cells/cm^2^ as described earlier^20^. Irradiation was performed in the exponential phase of cell growth at a dose rate of 19.16 Gy/min (Gamma Chamber 5000, Board of Radiation and Isotope Technology, Department of Atomic Energy, Mumbai, India) and cells were kept at 28 °C till harvesting.

### *In vitro* probe construction and microRNA identification

Construction of probe for the Sf-miR-31 identification was done using *Bombyx mori* mature miR-31 as a template for *in vitro* transcription. Mature miR-31 oligonucleotides (oligos) were synthesized in DNA format along with T7 promoter sequence (GGACAGAG) added at the 3′ end, which is complementary to the T7 promoter primer and facilitated hybridization of the two of DNA oligos so that the fill-in reaction produced a double-strand transcription template containing the T7 promoter site. Probes were constructed using miRNA probe construction kit (Ambion, Cat. No. AM1550) as per manufacturer’s protocol with some modifications, by adding 2 μl of T7 promoter primers, 6 μl of DNA hybridization buffer and 2 μl of oligonucleotide template and heating at 70 °C for 5 minutes followed by 5 minutes incubation at room temperature. Further, 2 μl of 10X Klenow reaction buffer, 2 μl of 10X dNTP mix, 4 μl of nuclease free water and 2 μl of Exo-klenow buffer were added and incubated at 37 °C for 45 min. Then 2 μl of this reaction was used for *in vitro* transcription by adding 2 μl of 10X transcription buffer, 1 μl each of ATP, CTP, GTP, biotin-labeled UTP, 2 μl of T7 RNA polymerase and RNase free water to make final volume to 20 μl. The reaction mix was kept at 37 °C for 30 min at RT, and DNase treatment was given to remove double stranded template. Thus formed biotin-labeled probe was used for the RNase protection assay. miRNA pool was isolated from Sf9 cells using *miR*Vana miRNA isolation kit (Ambion, Cat. No. AM1561), and mature miRNAs (<40bp) were screened using flash PAGE fractionators (Ambion, Cat. No. 13100) for hybridization with the biotinylated probes at 37 °C for 2 h. RNase protection assay was performed using *miR*Vana miRNA Detection Kit (Ambion, Cat. No. AM1552) as per manufacturer’s protocol. RNase-protected miRNAs/probe hybrids were then separated by 15% Urea-PAGE (8M Urea) and after transfer on to nylon membrane, detected with streptavidin-HRP.

### Western immunoblotting, sub-cellular fractionation and immunoprecipitation

For protein expression analysis, western immunoblotting was performed at different time intervals after treatment, as detailed earlier^25^. Sub-cellular fractionation was performed to observe the Bax translocation from cytosol to mitochondria using immunoblotting of both fractions. Both the fractions were separated using protocol as discussed in previous study^26^. Purity of cytosolic and mitochondrial fractions was confirmed using anti-VDAC and anti- GAPDH antibodies (Santa Cruz Biotechnology, Dallas, Texas, USA), respectively. For Bim-Bax interaction analysis, immunoprecipitation (IP) assay was done using anti-Bax antibody (Calbiochem, Sandiego, CA, USA). Reverse immunoprecipitation was also performed using anti –Bim antibody (Calbiochem, Sandiego, CA, USA) and detection by anti-Bax antibody. Cells were irradiated with and without previous transfection with AS-miR-31 and harvested 18 h post-irradiation. Cells were lysed using native lysis buffer (150 mM NaCl, 10 mM HEPES [pH 7.4], 1% CHAPS, 10% Protease inhibitor cocktail) and 250 μg of whole cell lysate was used for immunoprecipitation using Catch and Release kit (Millipore Cat. No. 17-500) as per manufacturer’s protocol. Eluted proteins were then separated by 15% PAGE and following transfer onto PVDF membrane, detected with anti-Bax and anti-Bim antibody (Calbiochem) and Enhanced Chemiluminescence (ECL) reagent (Pierce, Cat. No. 34087).

### Cell death/Apoptosis analysis

Cell death was assessed by observing cells under DIC microscope (Axiovert-200 Zeiss inverted DIC microscope, Carl Zeiss, Germany). Cell morphology assay^40^ and measurement of sub-G1 population by flow cytometry were used for determining dead cell population. In brief, cells were harvested 24h following irradiation with or without transfection with AS-miR-31. Direct cell suspension was used for embedding on agarose-laden slides and fluorescence staining with FITC-PI for assessing morphological changes. For flow cytometry, Cells were washed with PBS and fixed in 70% ethanol and kept for overnight incubation at −20 °C. Propidium iodide (20μg/ml) was used for nuclear staining following RNase treatment for 30 min at 37 °C. Relative DNA content and cell cycle distribution were analysed with the help of FACSCalibur flow cytometer (Becton- Dickinson & Co., New Jersey, USA) using the CellQuest software (v.3.0.1) for acquisition and ModFit LT software (v.2.0; Verity Software House, Inc., Maine, USA) for cell cycle analysis (Supplementary Fig. 1a). Again for more precise measurement of cell death, Sub-G1 cell population was gated and 5000 cells were counted in gated region irrespective of the total numbers of cells. Dead cells were then calculated by normalizing gated cells (5000 fixed number) against the total cell count (Supplementary Fig. 1b).

### Quantitative Real Time PCR and transfection

Radiation-induced changes in the expression of Sf-miR-31 were observed with real time PCR. miRNA isolation was performed using *miR*Vana microRNA isolation kit and mature miRNAs were screened using Flash PAGE Fractionators (Ambion). 1 μg of total miRNA was used for cDNA preparation in a 20 μl reaction using miScript Reverse Transcription Kit as per manufacturer’s protocol (Qiagen, Cat. No. 218061). 2 μl of cDNA was used to analyze the Sf-miR-31 expression using Sf-miR-31 specific forward primer in Real Time Thermocycler (Stratagene, MX3005P). Single peak of dissociation curve from the amplified produced was considered as specific amplification. For transfection of AS-miR-31 and mimic miR-31, 0.50 μg of each was used with RNAiFect (Qiagen, Cat No. 301605) in 1:5 ratio (w/v). The expression of miR-31 after antisense and mimic miR-31 was quantified using Real Time PCR (Supplementary Fig. 2). All the transfections were carried out in exponential phase of cell growth.

### Immunofluorescence microscopy and caspase activity assay

To study Bax translocation using immunofluorescence microscopy, cells were grown on cover slips in a 35 mm culture dish 24 h prior to transfection (with AS-miR-31 or MM-miR-31) and/or irradiation. Irradiated/treated cells were first incubated with mitotracker red (Life Technologies, Cat No. M-7512) for 30 min at 37 °C and washed twice with wash buffer containing PBS and Tween-20 (0.1% v/v). Cell fixation was done by incubating cells in chilled acetone for 10 min at 4 °C followed by permeabilization (0.05% TritonX-100 in PBS) for 5 min. Blocking was performed with 1% FBS in PBS for 30 min at RT to reduce nonspecific signals. Anti-Bax antibody was diluted in wash buffer at 1:100 dilution and incubated for 1 h at RT. After washing twice with wash buffer, cells were incubated with secondary antibody conjugated with FITC for 45 min, washed and mounted with 50% glycerol in PBS and images were captured using Leica SP8 spectral confocal microscope (Courtesy Leica Microsystems India). Caspase-3 activity was assessed 24h post-irradiation using caspase-3 activation assay kit (Sigma, Cat. No. CASP3F) according to manufacturer’s recommendation. The mean fluorescence values of triplicates were plotted to determine variation in caspase-3 activation.

### Target prediction/Gene Ontology term clustering

For target gene prediction of miR-31, we extracted 3′ UTRs of the *Bombyx mori* (silkworm, Dazao strain) from NCBI UniGene database (http://www.ncbi.nlm.nih.gov/sites/entrezdbunigene). These 3′ UTRs were then queried against *Bombyx mori* miR-31 (*Bmo*-miR-31) for target accessibility and interaction using PITA executable program^41^. We determined only those potential targets which had a score ΔΔG ≤ −15 kcal/mol (Supplementary Table 1). The locations of these screened UTR’s (nscaf) were then searched for the associated gene in the silkworm database. The reference sequence IDs (nucleic acid, protein) of those genes were then searched for the *Drosophila* homologs and converted to Flybase ID using DAVID Bioinformatics Resource, gene ID conversion tool^42^. As miRNA functions could be evolutionarily conserved between species such as *B. mori* and *D. melanogaster*^43^, we used Flybase IDs of identified targets for the GO term clustering using Panther and Cytoscape^44^ (Bingo) tools for the functional analysis of Sf-miR-31. Cytoscape (GeneMANIA) tool was used for the prediction of targets involved in stress signaling/cell death (Supplementary Fig. 3).

### Statistical Analysis

Differences between the mean values were analyzed for significance using the paired two tailed student’s ‘t’ test for independent samples using Microsoft Excel, with p value ≤ 0.05 considered as statistically significant.

## Additional Information

**How to cite this article**: Kumar, A. *et al*. Evidence for microRNA-31 dependent Bim-Bax interaction preceding mitochondrial Bax translocation during radiation-induced apoptosis. *Sci. Rep*. **5**, 15923; doi: 10.1038/srep15923 (2015).

## Supplementary Material

Supplementary Information

Supplementary Table S1
