# Supplementary material for: Evidence for microRNA-31 dependent Bim-Bax interaction preceding mitochondrial Bax translocation during radiation-induced apoptosis
Source: Sci Rep. 2015 Oct 30;5:15923. doi: 10.1038/srep15923 (PMC4626866; doi:10.1038/srep15923)
Supplement: Supplementary Information [file srep15923-s1.pdf]

Evidence for microRNA-31 dependent Bim-Bax interaction preceding mitochondrial Bax translocation during radiation-induced apoptosis

Ashish Kumar, Soma Ghosh and Sudhir Chandna*

Natural Radiation Response Mechanisms Group, Division of Radiation Biosciences, Institute of Nuclear Medicine & Allied Sciences, Brig. S.K. Mazumdar Road, Timarpur, Delhi-110054, India.

*To whom Correspondence should be addressed
Phone: + 91-011-23977027; Fax: +91-011-23919509
Email: sudhirchandna@yahoo.com
Current address:
Dr. Sudhir Chandna,
Natural Radiation Response Mechanisms Group, Division of Radiation Biosciences, Institute of Nuclear Medicine & Allied Sciences, Brig. S.K. Mazumdar Road, Timarpur, Delhi-110054, India.

Supplementary Fig. 1 (A)

Cell death measurement by flow cytometry

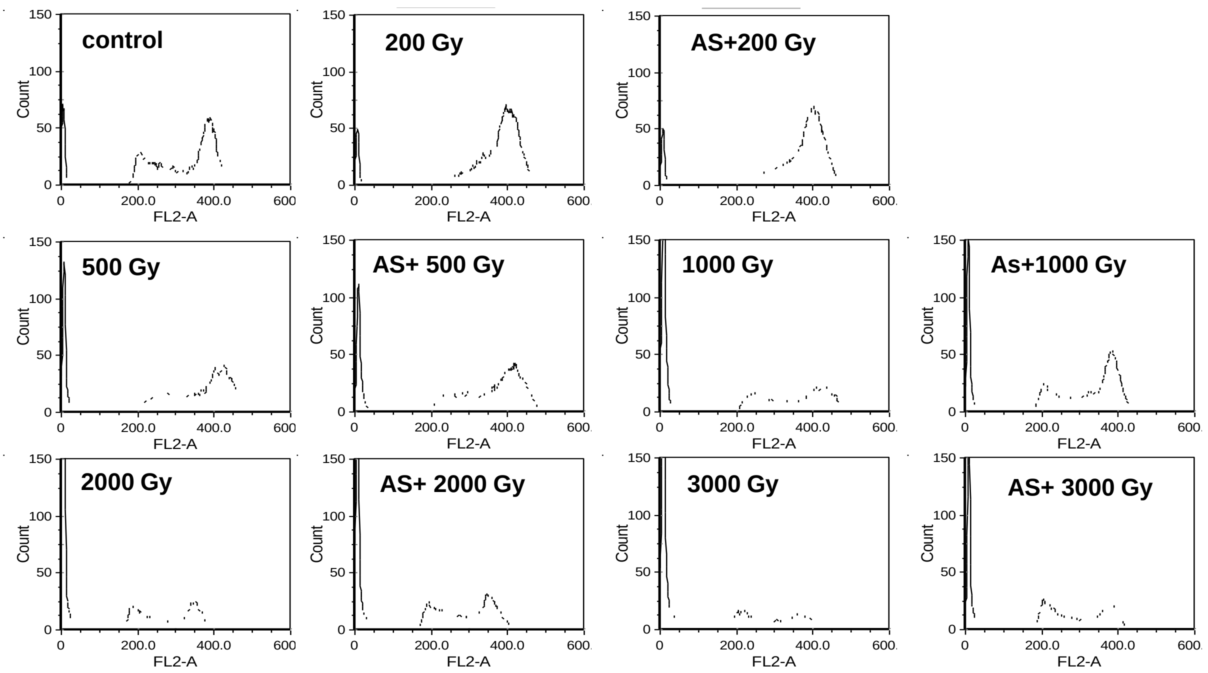

Supplementary Figure. 1 (B)

Dead Cell Measurement (5000 Fixed counts in gated sub-G1 population) by flow cytometry

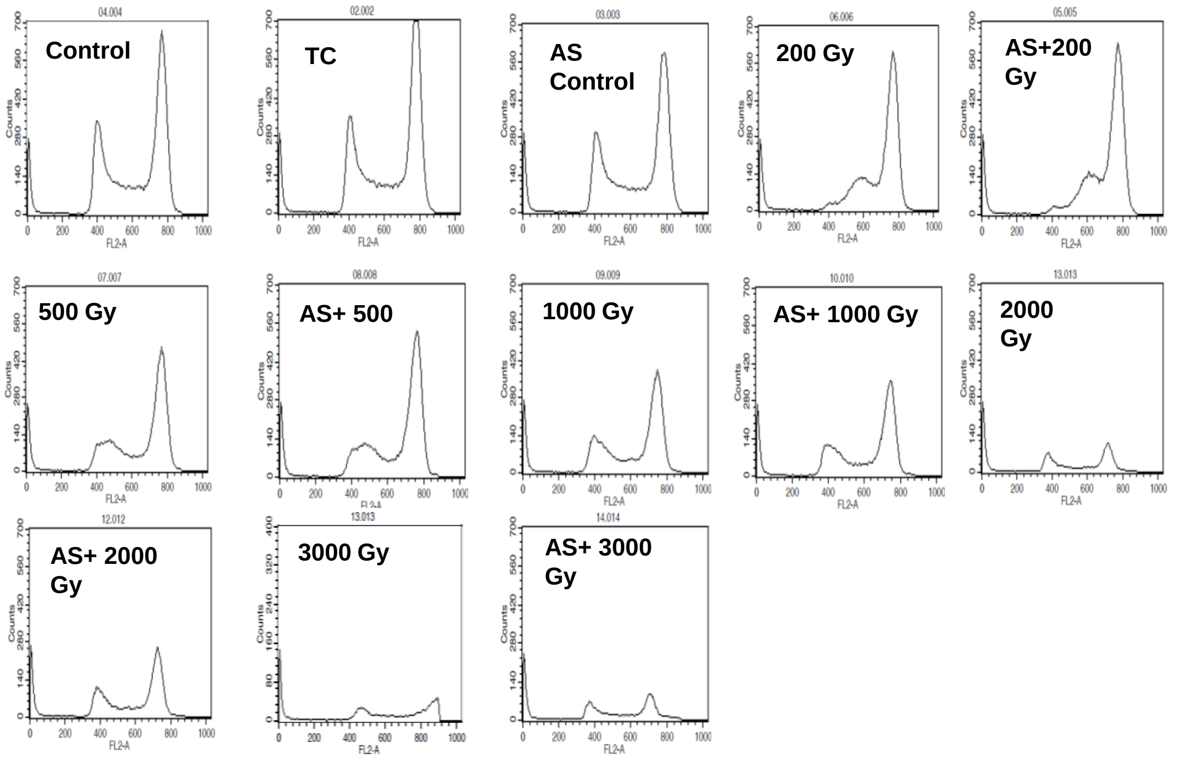

Supplementary Fig. 1 (A) Cell death measurement by flow cytometry after irradiation at different doses with or without pre-treatment with AS-miR-31. Analysis was done by measuring total SubG1 events as Sf9 cells always yield a debris population during mid-late stages of apoptosis (due to rapid DNA fragmentation), instead of a typical sub-G1 peak observed in mammalian cells. Transfection Reagent alone and with antisense did not show any effect on cell death (data not included). **(B)** Analysis was done by fixing 5000 counts for gated G1 subpopulation, irrespective of the total counts. Cell death was measured by normalizing total cell count against 5000 (fixed count). Transfection with AS-miR-31 showed no effect on cell cycle progression but a significant reduction was observed in cell death.

Supplementary Fig. 2

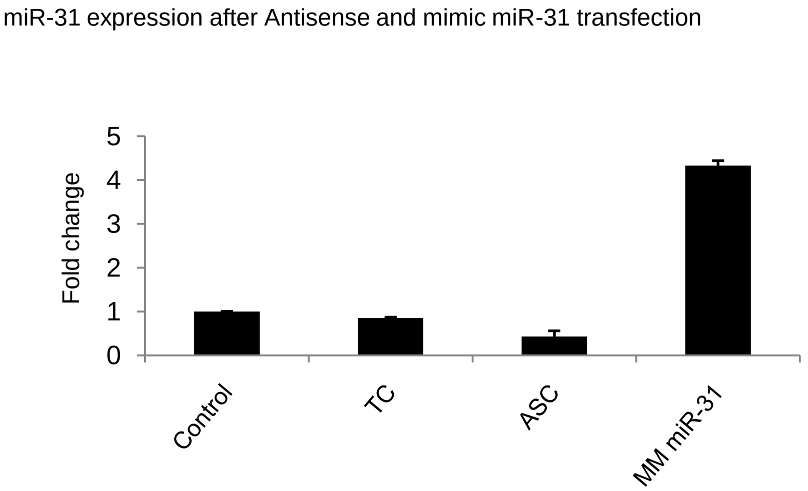

Supplementary Fig. 2 Expression of miR-31 was observed after transfection with antisense and mimic oligos of miR-31, 16-18 post-transfection by real time PCR analysis.

Supplementary Fig. 3

Candidate proteins involved in miR-31 dependent cell death regulation

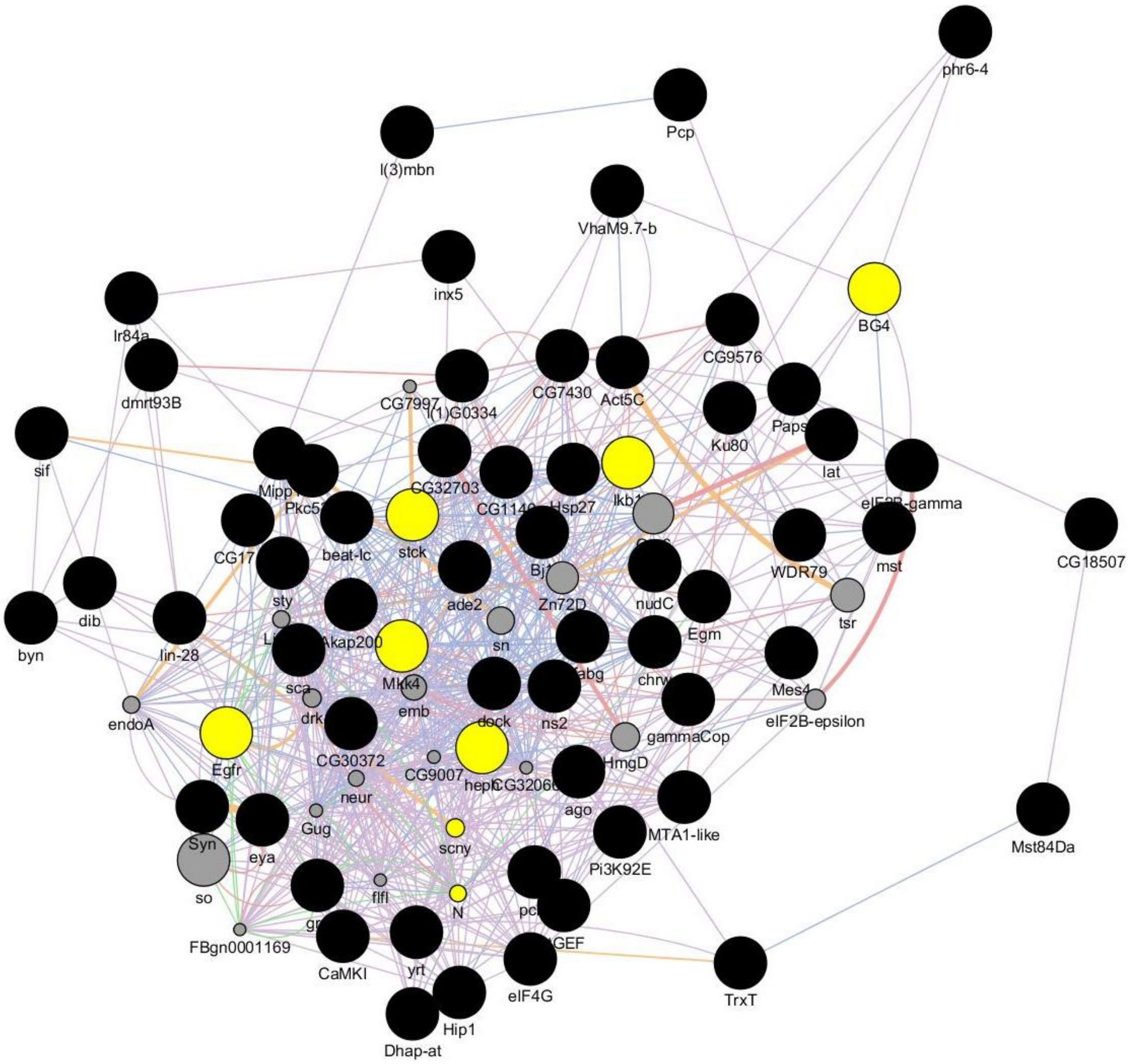

Supplementary Fig. 3 In silico prediction of Sf-mir-31 targets involved in apoptosis regulation. All the targets predicted for Sf-miR-31 were used for the Cytoscape (Genemania) analysis for the identification of genes involved in cell death regulation. Yellow marked genes represent those involved in regulation of apoptosis.
